# Collective Diffraction Effects in Perovskite Nanocrystal Superlattices

**DOI:** 10.1021/acs.accounts.2c00613

**Published:** 2022-12-19

**Authors:** Stefano Toso, Dmitry Baranov, Umberto Filippi, Cinzia Giannini, Liberato Manna

**Affiliations:** † Department of Nanochemistry, Istituto Italiano di Tecnologia, Via Morego 30, 16163 Genova, Italy; ‡ International Doctoral Program in Science, Università Cattolica del Sacro Cuore, 25121 Brescia, Italy; § Istituto Di Cristallografia − Consiglio Nazionale delle Ricerche (IC−CNR), I-70126 Bari, Italy

## Abstract

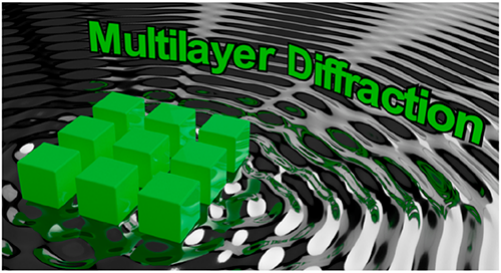

For almost a decade now, lead halide perovskite nanocrystals have been the subject of a steadily growing number of publications, most of them regarding CsPbBr_3_ nanocubes. Many of these works report X-ray diffraction patterns where the first Bragg peak has an unusual shape, as if it was composed of two or more overlapping peaks. However, these peaks are too narrow to stem from a nanoparticle, and the perovskite crystal structure does not account for their formation. What is the origin of such an unusual profile, and why has it been overlooked so far? Our attempts to answer these questions led us to revisit an intriguing collective diffraction phenomenon, known for multilayer epitaxial thin films but not reported for colloidal nanocrystals before. By analogy, we call it the multilayer diffraction effect.

Multilayer diffraction can be observed when a diffraction experiment is performed on nanocrystals packed with a periodic arrangement. Owing to the periodicity of the packing, the X-rays scattered by each particle interfere with those diffracted by its neighbors, creating fringes of constructive interference. Since the interfering radiation comes from nanoparticles, fringes are visible only where the particles themselves produce a signal in their diffraction pattern: for nanocrystals, this means at their Bragg peaks. Being a collective interference phenomenon, multilayer diffraction is strongly affected by the degree of order in the nanocrystal aggregate. For it to be observed, the majority of nanocrystals within the sample must abide to the stacking periodicity with minimal misplacements, a condition that is typically satisfied in self-assembled nanocrystal superlattices or stacks of colloidal nanoplatelets.

A qualitative understanding of multilayer diffraction might explain why the first Bragg peak of CsPbBr_3_ nanocubes sometimes appears split, but leaves many other questions unanswered. For example, why is the split observed only at the first Bragg peak but not at the second? Why is it observed routinely in a variety of CsPbBr_3_ nanocrystals samples and not just in highly ordered superlattices? How does the morphology of particles (i.e., nanocrystals vs nanoplatelets) affect the appearance of multilayer diffraction effects? Finally, why is multilayer diffraction not observed in other popular nanocrystals such as Au and CdSe, despite the extensive investigations of their superlattices?

Answering these questions requires a deeper understanding of multilayer diffraction. In what follows, we summarize our progress in rationalizing the origin of this phenomenon, at first through empirical observation and then by adapting the diffraction theory developed in the past for multilayer thin films, until we achieved a quantitative fitting of experimental diffraction patterns over extended angular ranges. By introducing the reader to the key advancements in our research, we provide answers to the questions above, we discuss what information can be extracted from patterns exhibiting collective interference effects, and we show how multilayer diffraction can provide insights into colloidal nanomaterials where other techniques struggle. Finally, with the help of literature patterns showing multilayer diffraction and simulations performed by us, we demonstrate that this collective diffraction effect is within reach for many appealing nanomaterials other than halide perovskites.

## Key References

- TosoS.; BaranovD.; GianniniC.; MarrasS.; MannaL.Wide-Angle X-ray Diffraction Evidence of Structural Coherence in CsPbBr_3_ Nanocrystal Superlattices. ACS Mater. Lett.2019, 1, 272–27610.1021/acsmaterialslett.9b0021732954357PMC7497715(1) Explanation of the first Bragg peak splitting in the diffraction pattern of CsPbBr_3_ nanocubes. Determination of the superlattice periodicity. Demonstration of the diffraction profile formation during the superlattice growth and of its *in situ* evolution during the superlattice contraction under vacuum.
- TosoS.; BaranovD.; AltamuraD.; ScattarellaF.; DahlJ.; WangX.; MarrasS.; AlivisatosA. P.; SingerA.; GianniniC.; MannaL.Multilayer Diffraction Reveals That Colloidal Superlattices Approach the Structural Perfection of Single Crystals. ACS Nano2021, 15, 6243–625610.1021/acsnano.0c0892933481560PMC8155329(2) Multilayer diffraction fit of patterns from CsPbBr_3_ and PbS nanocrystal and nanoplatelet superlattices. Determination of nanocrystal thickness, interparticle distance, and average nanocrystal displacement. Measured displacement parameters ≤1 Å are evidence of nearly single-crystal perfection for colloidal nanocrystal superlattices.
- TosoS.; BaranovD.; GianniniC.; MannaL.Structure and Surface Passivation of Ultrathin Cesium Lead Halide Nanoplatelets Revealed by Multilayer Diffraction. ACS Nano2021, 15, 20341–2035210.1021/acsnano.1c0863634843227PMC8717630(3) Further development of the multilayer diffraction model to include an atomistic description of nanocrystals. Application to Cs–Pb–X nanoplatelets with perovskite and Ruddlesden–Popper structures to refine surface passivation, defects, and structure distortions as compared to the corresponding bulk analogs.

## (Re)discovery of Multilayer Diffraction

**Figure 1:**
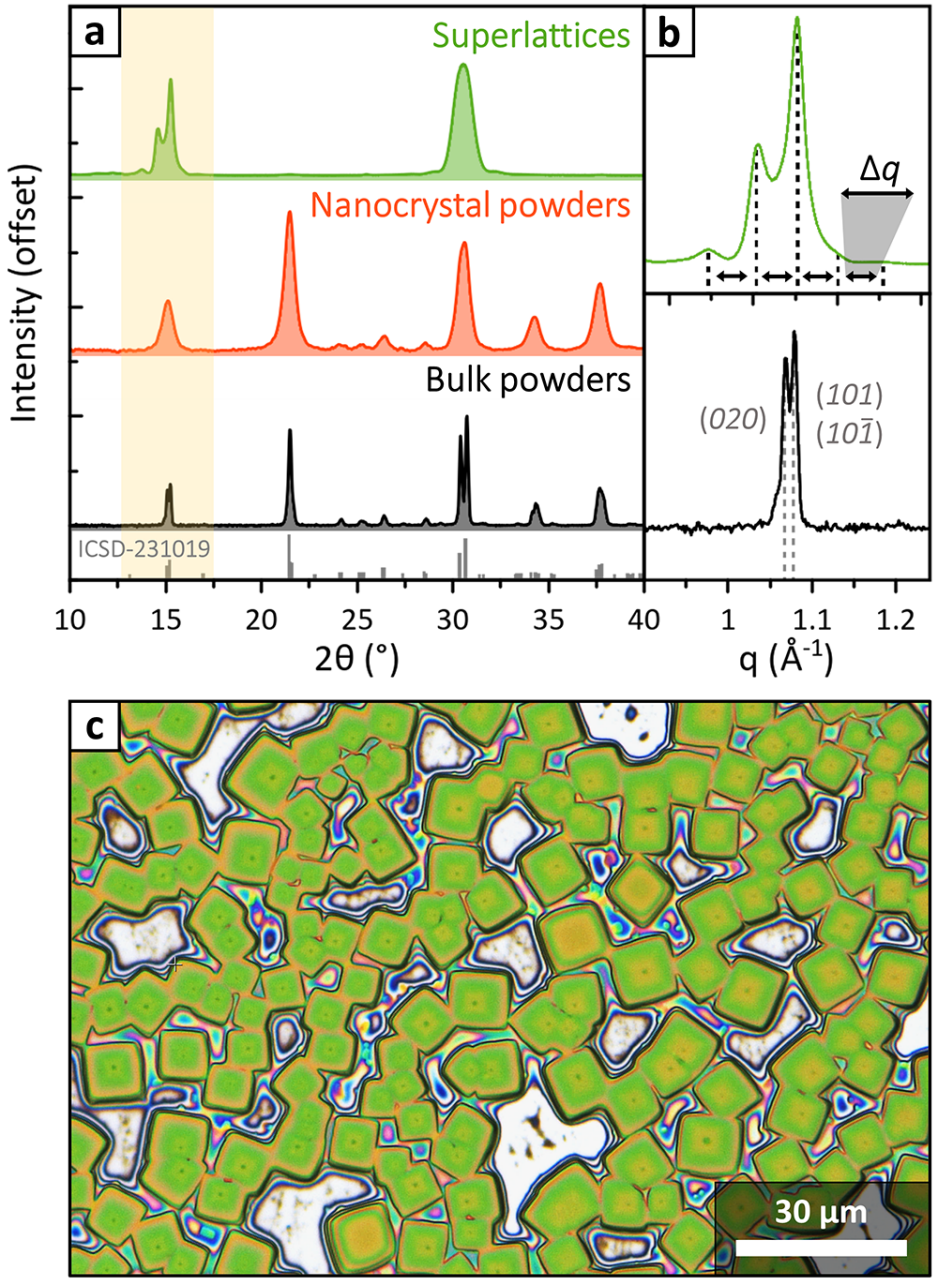
XRD patterns of different CsPbBr_3_ samples. (a) XRD patterns of CsPbBr_3_ superlattices (green), nanocrystal powders (red), and bulk powders (black, orthorhombic *Pnma* reference in gray).^6^ Peaks from nanocrystal powders retain the positions and relative intensities of the bulk but are broadened due to the nanometric size. In the superlattices pattern most peaks are suppressed due to preferred orientation, and the first Bragg peak (yellow shaded area) is visibly split. (b) Close-up of the first superlattice peak plotted on the *q* scale (top). Superlattice fringes are sometimes confused with the cubic (100) → orthorhombic (020)/(101)/(101̅) CsPbBr_3_ peak split, shown on the bulk pattern for comparison (bottom). (c) Optical microscopy image of CsPbBr_3_ nanocube superlattices. Adapted with permission from refs (1) and (2). Copyright (2019, 2021) American Chemical Society.

The (re)discovery of multilayer diffraction in colloidal superlattices was due to chance. In 2019, we were investigating the spectral properties of CsPbBr_3_ nanocrystal solids grown at a liquid–liquid interface,^4^ an approach that, albeit effective, yielded samples that were hard to manipulate. Therefore, we started growing superlattices on silicon substrates, which were more versatile for a variety of experiments. One of them was X-ray diffraction (Figure 1), which we first performed to monitor the sample stability. Based on prior works on nanocrystal assemblies,^5^ we expected to observe few broad reflections selected by the nanocrystals’ orientation, as only lattice planes parallel to the substrate would produce signals. Indeed, only two of the CsPbBr_3_ Bragg peaks were observed, but, surprisingly, one was noticeably split into fringes (Figure 1a).^1^

If interpreted through the Scherrer equation,^7^ the width of such fringes would indicate a ∼37 nm crystal size, much larger than the ∼10 nm CsPbBr_3_ nanocubes we self-assembled. Moreover, we observed that fringes shifted and changed their intensities upon exposing the sample to vacuum, but the sample itself did not suffer any other visible alteration, suggesting that their appearance cannot be simply ascribed to the crystal structure of nanoparticles. A first hint of their origin came from plotting the pattern on the scattering vector scale *q* = 4π sin (θ)/λ_X-ray_. This revealed that fringes were regularly spaced in *q*, compatibly with a real-space periodicity of Λ = 2π/Δ*q* = 12.2 nm, where Δ*q* is the distance between fringes (Figure 1b). Such a length matched the nanocrystals’ center-to-center distance measured by electron microscopy,^1^ persuading us that the Bragg peak split was not due to changes in the structure of nanocrystals but rather due to their regular packing within the superlattice.

Indeed, we soon learned that similar fringes are commonly observed in multilayer epitaxial thin films composed of neatly stacked crystalline layers, where they are called satellite peaks. These layered materials have been studied since the 1980s, and many theoretical descriptions for their diffraction patterns were developed over the years.^8−15^ In what follows, we picked the formalism developed by Schuller and colleagues,^16−19^ whose modular nature makes it easy to tune, and we adapted it to describe superlattices of colloidal nanocrystals. We find it fascinating that 40 years after coherent diffraction was first observed on epitaxial films the same effect could be revisited in a remarkably different system as colloidal nanocrystal superlattice.

## Principles of Multilayer Diffraction

**Figure 2:**
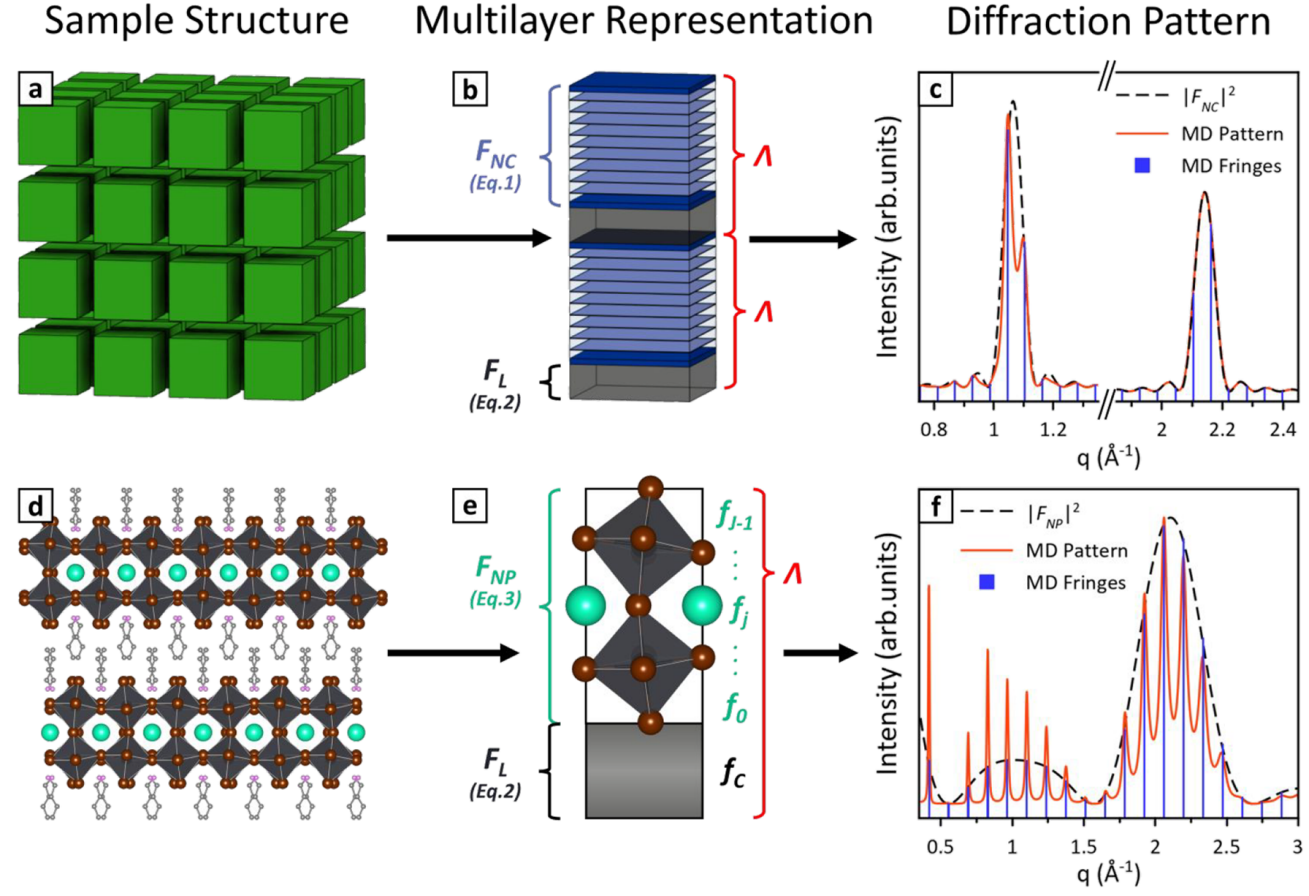
Principles of multilayer diffraction. A nanocrystal superlattice (a) is composed of (b) alternating inorganic (blue) and organic layers (black), both contributing to the superlattice periodicity Λ (red). Each layer has a scattering factor for nanocrystals (*F*_NC_) and for organic ligands (*F*_L_), computed via eqs 1 and 2 respectively. A multilayer diffraction pattern (c, red line) stems from the constructive interference of many such organic–inorganic bilayers. Fringes are affected by the superlattice periodicity (position, *q*_*n*_ = 2π*n*/Λ), superlattice disorder σ_L_ (width), and scattering factors (intensity). *F*_NC_ is predominant due to the high electron density in nanocrystals, resulting in the intensity of fringes being modulated by the nanocrystal Bragg peaks (c, black dashed line). Similarly, a stack of nanoplatelets (d) is a multilayer (e) of alternating inorganic (cyan) and organic layers (black), resulting in the nanoplatelet scattering factor *F*_NP_ modulating the intensity of the multilayer diffraction fringes (f). The only difference between (a–c) and (d–f) is that *F*_NC_ is computed based on unit cells, while for *F*_NP_ one must consider individually each atomic layer within the platelet (*f*_0_*... f*_*J*–1_). Adapted with permission from refs (2) and (3). Copyright (2021) American Chemical Society.

We begin by providing a general overview of multilayer diffraction (Figure 2), while its mathematical aspects are covered in detail elsewhere.^16−19^ The ideal experiment for collecting multilayer diffraction data is a symmetric out-of-plane θ:2θ scan. This is one of the most common diffraction experiments, often used to characterize powder and thin film samples. The fundamental property of such geometry is that the scattering vector *q* is perpendicular to the sample surface (see SI, section S1). This condition allows us to describe the sample as a vertical stack of planes, disregarding its in-plane structure, and justifies the use of a multilayer-based approach to model a superlattice diffraction pattern. Interference fringes might be observed in other geometries as well, but the model we introduce here would not provide a quantitative description of the diffracted intensity.

Multilayer diffraction occurs only where radiation is available to interfere in the first place. This is provided by the two superlattice components, namely nanocrystals and organic ligands, that act as radiation sources by diffracting the incident beam. Nanocrystals are the electron-dense part of the superlattice and therefore provide most of the diffracted intensity. This is encoded in their scattering factor *F*_NC_(*q*), which is the amplitude of the electric field diffracted by one nanocrystal at each *q* (or 2θ) value. For nanocrystals of generic shape diffracting with their (*hkl*) planes, *F*_NC_(*q*) is the sum in phase of radiation scattered by each unit cell plane *n* (eq 1).
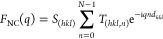
1Here, *S*_(*hkl*)_ is the structure factor of a unit cell oriented in the [*hkl*] direction, *d*_(*hkl*)_ is the periodicity of (*hkl*) planes, *T*_(*hkl*,*n*)_ describes the nanocrystal shape through the number of unit cells per plane (see SI, section S2), and *N* is the nanocrystal thickness through the number of unit cell planes. Depending on circumstances, eq 1 can be simplified: for example, *T*_(*hkl*,*n*)_ is constant for cube-shaped nanocrystals with their facets parallel to the substrate, as in CsPbBr_3_ superlattices. Moreover, if we analyze one Bragg peak at a time, then the relative intensity of peaks becomes irrelevant and *S*_(*hkl*)_ can be considered constant.

Organic ligands are the electron-sparse component of the superlattice. Their contribution to the diffracted intensity is often negligible [*F*_L_(*q*) ≈ 0], except for samples with a large organic/inorganic volume ratio, such as stacks of thin nanoplatelets. In this case, *F*_L_(*q*) can be approximated to the scattering of an amorphous carbon layer (eq 2)
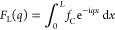
2where *f*_C_ is the atomic scattering factor of carbon and *L* is the organic layer thickness. Note that eq 2 resembles eq 1 except for the nature of scatterers (atoms vs unit cells) and for their continuous vs discrete spatial distribution.

Together, nanocrystals and ligands alternate with an overall periodicity Λ, which is captured by the position of the diffraction fringes according to the relation Λ = 2π/Δ*q*. Deviations from the ideal periodicity Λ represent a form of disorder and can have a major impact on multilayer diffraction. Indeed, each layer can be subject to a misplacement that breaks down into two contributions: a discrete disorder σ_*N*_ due to the nanocrystal size distribution and a continuous stacking disorder σ_*L*_ due to inhomogeneities in the ligand layer.

Because their thickness is a multiple of *d*, nanocrystals larger or smaller than the average will misplace the layers above them by a factor of *nd* (hence the “discrete” labeling for σ_*N*_). However, a shift matching the periodicity of atomic planes does not disrupt the interference in correspondence of Bragg peaks, making the effect of discrete disorder negligible for samples with narrow size distributions.

Conversely, the stacking disorder σ_*L*_ bears central importance in multilayer diffraction. This is caused by random fluctuations in the thickness of the organic layers due to their soft and noncrystalline nature, which is described by a Gaussian distribution centered around *L* and with standard deviation σ_*L*_.^17−19^ Crucially, the disorder in superlattices is cumulative, as one misplaced layer will shift all those above: the accumulation of multiple random misplacements results in the broadening of diffraction fringes at higher *q* values and eventually causes them to fade completely.^17−19^ That is why CsPbBr_3_ nanocube superlattices display a peak split only at the first Bragg peak (*q* ≈ 1 Å^–1^) but not at the second (*q* ≈ 2 Å^–1^).

It follows that a low stacking disorder is mandatory to observe multilayer diffraction. As a rule of thumb, σ_*L*_ ≤ 1 Å would allow to observe interference up to ∼20° 2θ_Cu Kα_ (*q* ≈ 1.5 Å^–1^). This is both a strength and a weakness of multilayer diffraction: on the one hand it is limited to highly ordered systems, but on the other hand such sensitivity enables high precision in quantifying the disorder. The most remarkable conclusion, however, is that colloidal superlattices can achieve the same structural perfection as materials grown epitaxially. For example, in CsPbBr_3_ superlattices, σ_*L*_ ≈ 1 Å, which is only ∼1% of Λ ≈ 100 Å and much shorter than a Pb–Br bond (∼3 Å). For comparison, some of the epitaxial films studied in refs (2), (17), and (19) had σ_*L*_ = 1.4 Å.

## The Case of Platelets

Compared to isotropic nanocrystals such as cubes or spheres, nanoplatelets demonstrate much stronger multilayer diffraction effects (Figure 2). First, nanoplatelet stacks are typically more ordered thanks to their anisotropic shape and strong face-to-face interactions. For example, lead halide nanoplatelets can reach σ_*L*_ values that are twice as low as those of nanocubes (σ_*L*_ ≈ 0.5 Å).^2,3^ Moreover, due to their extreme thinness nanoplatelets do not produce well-defined Bragg peaks but rather a broad and continuous diffraction profile. Hence, multilayer diffraction is observed over a much wider angular range: while nanocrystal superlattices are usually limited to ∼3–5 fringes per Bragg peak (Figure 2a–c),^3^ nanoplatelet stacks often display up to ∼20 fringes in total (Figure 2d–f).^2^

Multilayer diffraction is so common for nanoplatelets that interference fringes are routinely exploited to extract their stacking periodicity (Λ = 2π/Δ*q*): an early example for perovskite nanoplatelets was reported by Weidman et al.^20^ However, the intensity of fringes is more challenging to describe than in nanocrystals. Due to the sudden crystal truncation and surface termination effects, nanoplatelets are not described properly by a unit cell and its associated *S*_(_*_hkl_*_)_.^3^ Hence, eq 1 must be replaced in favor of an atomistic description (eq 3).
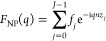
3Here, *f*_*j*_ is the atomic scattering factor of the *j*th atom, *J* is the number of atoms in the platelet, and *z*_*j*_ is their vertical coordinate. In principle, *J* would be a very large number. However, atoms of the same element and belonging to the same plane give identical contributions. Therefore, the summation can be limited to the handful of atoms needed to capture the nanoplatelet stoichiometry, greatly simplifying its description. Note that eq 3 could be used to describe nanocrystals as well, replacing eq 1. This, however, would be impractical, as the number of atomic planes increases quickly with the nanocrystal thickness.

## Why Perovskite Nanocrystals?

The literature on metal halide nanocrystals is rich with XRD patterns showing Bragg peaks split in fringes^21−29^ or having asymmetric shapes,^30−33^ which are typical signatures of multilayer diffraction. However, only a few of these studies concern nanocrystal superlattices (refs (21) and (25−28)), while most did not target or mention the formation of self-assembled structures. On the other hand, multilayer diffraction was not reported for any of the highly ordered superlattices that have been grown for decades from nanocrystals of other materials. What makes perovskites so suitable for observing this effect?

Having constructed a picture of multilayer diffraction, we can now explain the factors contributing to this apparent contradiction. A first element is the sample preparation. Nanocrystal samples for XRD are often prepared by drop casting nanocrystal dispersions on a flat substrate. While drying, nanocrystals might form self-assembled domains, which would result in the appearance of multilayer diffraction fringes even when they are only a few nanocrystals thick, provided that the internanocrystal distance is consistent across different domains (see SI, section S3).^2^ This explains why fringes are seen even in samples not recognized as superlattices.^2^ One of the first clues that superlattices might have formed upon drop casting is preferred orientation, which is revealed by the suppression or weakening of some diffraction peaks and is indeed visible in refs (21−33).

Second, we consider the nanocrystal shape and structure. Multilayer diffraction is challenged by the stacking disorder, which disrupts the fringes starting from higher angles. Here, lead halide perovskite nanocubes have a twofold advantage. Their first Bragg peak falls at lower angles (2θ_Cu Kα_ = 15°) than those of other popular superlattice materials such as Au (2θ_Cu Kα_ = 38°), PbS (2θ_Cu Kα_ = 26°), or CdSe (2θ_Cu Kα_ = 24°). This relaxes the σ_*L*_ requirements, making perovskite superlattices more prone to display multilayer diffraction. Moreover, the cubic shape of perovskite nanocrystals makes them assemble in the correct orientation for observing such a peak. As a counterexample, the lowest-angle peak of PbS is (111), but nanocubes in a simple cubic arrangement would orient so that the higher-angle (200) peak is measured instead. Finally, cube-shaped nanocrystals favor a lower stacking disorder compared to spheroidal nanocrystals, as the simple cubic packing is more compact and constrained than the sparser BCC, FCC, and HCP geometries typical of spheres.

Third, nanocrystal superlattices are traditionally studied by grazing incidence small-/wide-angle X-ray scattering (GISAXS/GIWAXS). The first operates in the small-angle regime, where diffraction comes from the nanoscale electron density modulation of the entire superlattice. Multilayer diffraction in the form we are discussing cannot occur there, as it is a secondary interference phenomenon building upon radiation diffracted by nanocrystals at wide angles. GIWAXS instead is a wide-angle technique, so it might in principle detect such interference. However, current GIWAXS instruments tend to have a worse angular resolution than instruments operating in a θ:2θ geometry, which could end up hiding interference fringes should they form. Moreover, GIWAXS data are often presented as two-dimensional maps, while multilayer diffraction is better seen by integrating data in slices. We therefore suspect that multilayer diffraction went unnoticed because θ:2θ scans, which would give the best chances of observing interference fringes, are seldom performed on superlattices in favor of grazing incidence techniques.

Finally, one might wonder why we first recognized multilayer diffraction in nanocubes, despite interference fringes being ubiquitous and much stronger in nanoplatelets.^20,34−39^ One reason is that the XRD patterns of nanoplatelet stacks closely resemble those of layered bulk materials such as Ruddlesden–Popper perovskites and are often rationalized by this analogy. Indeed, most works on perovskite nanoplatelets acknowledge that fringes are due to their stacking, but then assign them Miller indices by analogy to the Bragg peaks of a Ruddlesden–Popper bulk crystal.^34,39,40^ This diverts attention from asking why bulk-like peaks are observed in a colloidal system in the first place and why their intensity appears to be modulated over a broader profile, two key questions that could have led to the identification of a multilayer interference effect.

## How Can Multilayer Diffraction Be Used?

**Figure 3:**
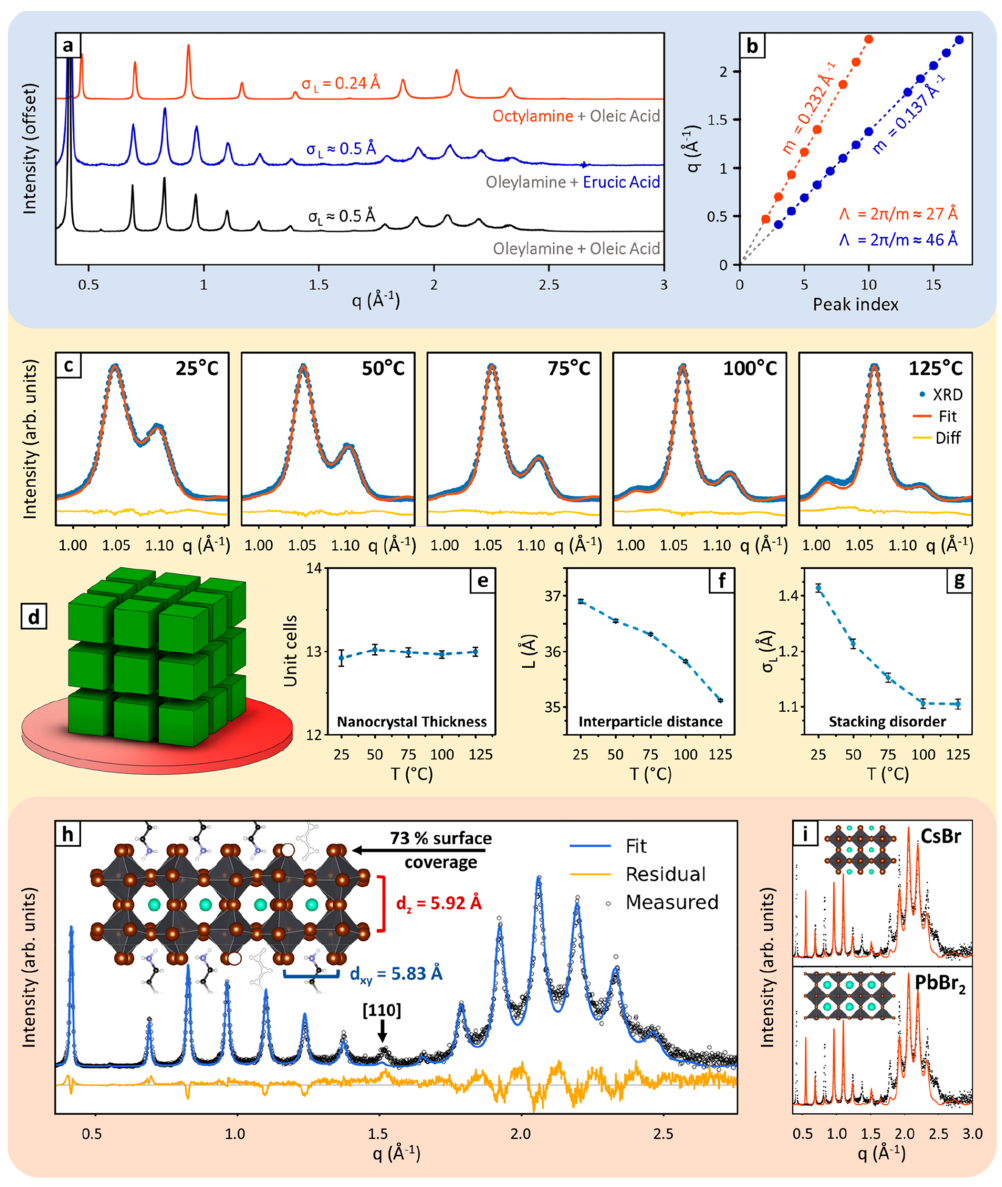
Multilayer diffraction for nanoparticle characterization. (a) Comparing patterns of samples prepared with different ligands can give insight into the surface passivation of nanocrystals. Replacing the carboxylic acid in the synthesis of perovskite nanoplatelets leaves their pattern unchanged, while different amines result in different stacking periodicities, demonstrating that platelets are passivated by amines only. (b) Linear regression allows the extraction of *Δq* and therefore Λ. (c) Evolution of the first Bragg peak of CsPbBr_3_ nanocube superlattices upon thermal annealing (d). (e–g) Evolution of nanocrystal thickness, interparticle distance, and stacking disorder σ_*L*_ tracked by multilayer diffraction. (h, i) The surface termination of perovskite nanoplatelets is identified by comparing their diffraction pattern with simulations. CsBr and PbBr_2_ terminations (i) are excluded due to mismatching fringe intensities, while an oleylammonium bromide termination (h) matches the pattern. (h) A quantitative profile fit allows refining the vertical atomic coordinates and the surface coverage factor. Adapted with permission from refs (2) and (3). Copyright 2021 American Chemical Society.

When present, multilayer diffraction is a powerful tool for studying nanocrystal assemblies. On the one hand, it provides insights into their nanoscale periodicity and disorder without the need of specialized instrumentation. On the other hand, being a wide-angle technique, it also provides information about the atomic structure of nanocrystals. Below, we present examples illustrating these points (Figure 3).

### Determination of Λ

The superlattice periodicity Λ is straightforward to extract: convert the position of fringes from 2θ to *q* (*q* = 4π sin (θ)/λ_X-ray_), assign a progressive index to each fringe, and perform a linear regression to extract the slope Δ*q* = 2π/Λ. To help resolve closely spaced fringes, the diffraction profile may be decomposed into a sum of Gaussians. This is the most accessible application of multilayer diffraction, as it does not require modeling the pattern. By this approach, we monitored the contraction of CsPbBr_3_ nanocrystal superlattices under vacuum due to the desorption of residual solvents and free ligands.^1^ Similarly, we could also track the structural changes in CsPbBr_3_ nanocrystal superlattices upon aging^27^ and in mixed-halide CsPb(I_1−*x*_Br_*x*_)_3_ nanocrystal superlattices under UV light.^28^ In the first case, the progressive fading of fringes and the appearance of sharp reflections typical of bulk CsPbBr_3_ allowed us to track the degradation of superlattices. In the second case, the illumination induced the expulsion of iodine, causing a contraction of the nanocrystal unit cell that eventually reduced the superlattice periodicity.

Comparing Λ between different samples can be a strategy for studying the nanocrystal surface. For example, lead halide nanoplatelets are often synthesized in the copresence of amines and carboxylic acids as surfactants. To identify which one passivates the surface, we compared Λ of a sample prepared with oleylamine and oleic acid with two other samples: one with a shorter amine (octylamine) and one with a longer carboxylic acid (erucic acid).^3^ While the erucic acid left Λ unchanged, the octylamine resulted in its drastic contraction, demonstrating that perovskite nanoplatelets are passivated by amines only (Figure 3a).

### Estimation of Stacking Disorder

The stacking disorder σ_*L*_ is proportional to the width of interference fringes, and can be extracted by fitting the experimental pattern with the open-source Python scripts we developed for nanocrystal superlattices and nanoplatelet stacks.^2,3^ Like Λ, σ_*L*_ helps in comparing samples and treatments. For example, mild thermal annealing (Figure 3c–g) improves the stacking order in CsPbBr_3_ nanocube superlattices. Instead, for nanoplatelets the stacking order is drastically improved by replacing oleylamine with octylamine, highlighting the importance of ligand engineering for optimizing nanocrystal assemblies (Figure 3a). The need for a profile fit makes σ_*L*_ less accessible than Λ. However, disorder in two different samples can be compared by the width of interference fringes, while a numerical estimate is obtained by observing at which *q* value the interference fringes fade out (eq 4).^2^
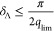
4Here, *q*_lim_ is the last *q* value at which fringes are observed. Note that δ_Λ_ is not equivalent to the more rigorously defined σ_*L*_, which should always be preferred when obtainable, but it provides a reasonable estimate. For example, CsPbBr_3_ nanocube superlattices show fringes at their first Bragg peak (Figure 1b, *q* ≈ 1 Å^–1^) but not at the second (*q* ≈ 2 Å^–1^), corresponding to 0.7 Å < δ_Λ_ ≤ 1.6 Å. Indeed, σ_*L*_ falls in the range of 1–1.5 Å (Figure 3g). Instead, for perovskite nanoplatelets the fringes can be observed up to *q* ≈ 2.5 Å^–1^ (Figure 3a), corresponding to δ_Λ_ ≤ 0.4 Å^–1^. Indeed, fitting the experimental patterns yielded σ_*L*_ values in the range of 0.25*–*0.5 Å^–1^.

### Nanoparticle and Organic Layer Thicknesses

Fitting the XRD pattern allows dividing Λ into nanoparticle and organic layer thicknesses. In fact, the nanoparticle thickness affects its scattering factor according to eqs 1 and 3, and is reflected in the number and intensity of visible fringes. Once both Λ and the nanoparticle thickness are known, the organic layer thickness is determined by difference. For example, on CsPbBr_3_ superlattices the fit allowed the measurement of the average nanocrystal size down to the single unit cell (Figure 3e) and enabled tracking the contraction of interparticle spacing during a thermal annealing experiment (Figure 3f). For nanoplatelets, the sensitivity is even higher, as adding or removing a single atomic plane substantially alters the diffraction profile. For example, for Cs–Pb–Br nanoplatelets we measured a thickness of 11.84 Å and an interplatelet distance of 34.0 Å (Figure 3h), in excellent agreement with the reported thickness of pure oleylamine lipid bilayers (3.4 nm).^41^ Here, multilayer diffraction provides substantial advantages over other techniques, as it ensures a direct and precise measurement of two parameters. Conversely, it is common to measure Λ by diffraction or TEM and then simply assume an approximate value for the nanocrystal thickness (for perovskites, generally a multiple of 0.6 nm)^40,42^ or the interparticle distance (for oleylamine, generally ∼2 to 3 nm)^27,37,43−45^ to estimate the counterpart by difference, leading to imprecise results.

### Atomic Structure Identification and Refinement

The structure of nanocrystals plays a central role in multilayer diffraction, as through eqs 1 and 3 it determines the diffraction profile that convolutes the intensity of interference fringes. This marks the distinction with small-angle techniques such as GISAXS, where the diffracted intensity comes from the nanoscale electron density modulation of the superlattice. Due to the θ:2θ diffraction geometry, multilayer diffraction is sensitive only to the vertical position of atoms and is therefore unable to determine the atomic coordinates in all other directions. Hence, powder XRD experiments followed by Rietveld or total scattering analyses are much better suited for refining the structure of nanocrystals.^46−48^

However, multilayer diffraction offers a significant advantage for very thin nanoplatelets. Here, the disappearance of Bragg peaks makes Rietveld refinement inapplicable, and total scattering methods might struggle as well, as nanoplatelets in powder or suspension might bend and curl, causing severe crystal structure deformations that are challenging to model. Indeed, the thinnest perovskite nanoplatelets refined by such methods were relatively thick (3.5 nm) and hence much more rigid.^49^ Conversely, the neat stacking achieved in nanoplatelet multilayers ensures that all particles are optimally aligned and flat. Therefore, a multilayer diffraction full-profile fit is a unique opportunity to validate the structural model of nanoplatelets down to the single atomic plane. For example, comparing experimental data with simulated diffraction profiles allowed us to determine unambiguously that Cs–Pb–Br nanoplatelets are passivated by oleylammonium bromide, ruling out competing PbBr_2_ or CsBr terminations (Figure 3h,i). Moreover, by leaving the layer occupancies and vertical coordinates as fittable parameters (Figure 3h), we could refine accurately the surface coverage (73%), the thickness (11.84 Å), and the degree of octahedra tilting in nanoplatelets.

## Prospective Applications

**Figure 4:**
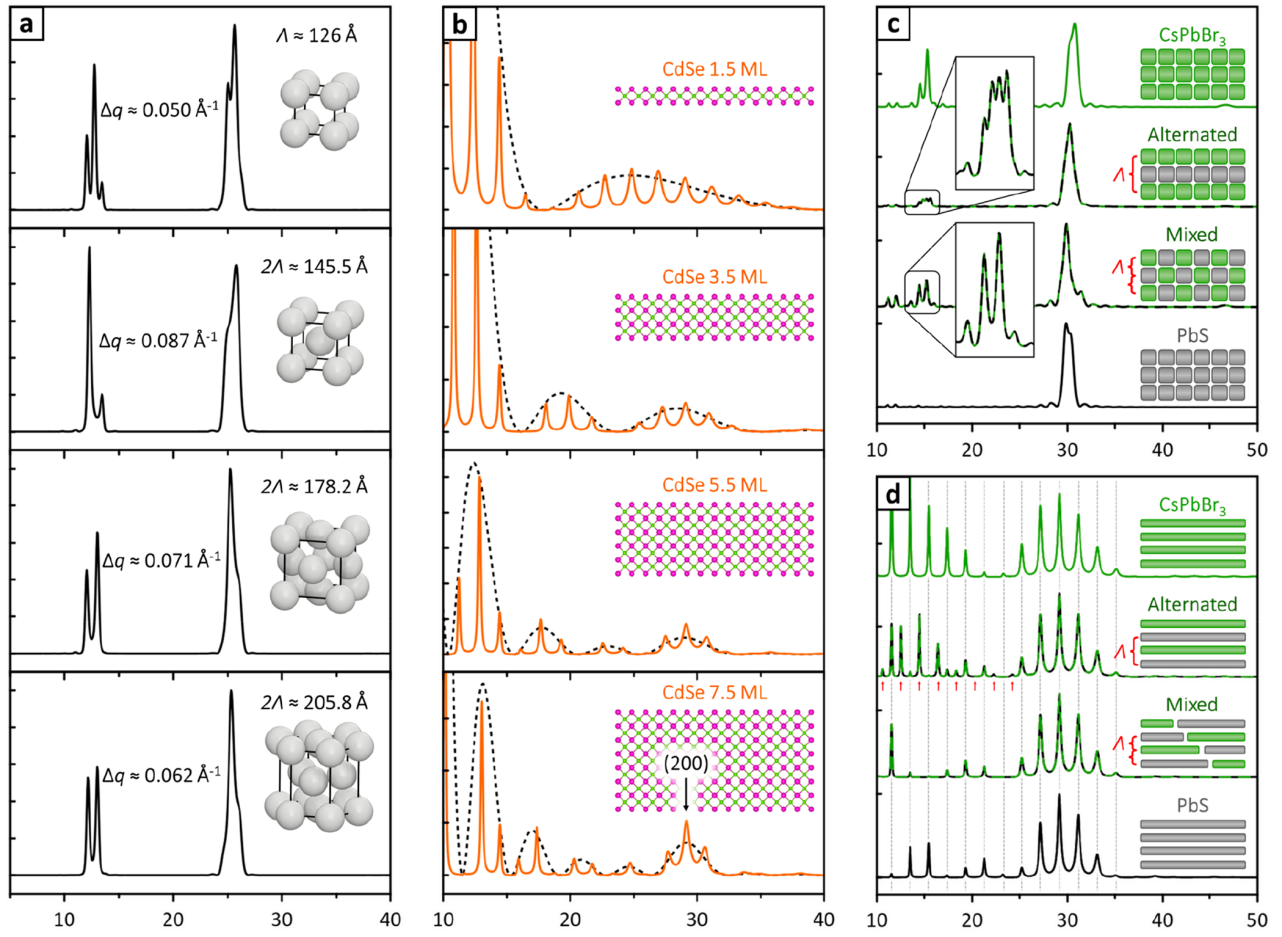
Simulated multilayer diffraction patterns. (a) Cs_4_PbBr_6_ nanospheres oriented with (012) planes parallel to the substrate and packed in different geometries. From top to bottom: simple cubic, BCC, FCC, and HCP. (b) Sphalerite CdSe nanoplatelets of different thicknesses, indicated as Cd–Se monolayers (ML). The diffraction profile of a single nanoplatelet (= |*F*_NP_|^2^) is traced by a black dashed curve. (c) Simple cubic superlattices of CsPbBr_3_ and PbS nanocubes and their mixtures. (d) Stacks of CsPbBr_3_ and PbS nanoplatelets and their mixtures. If two kinds of nanocrystals do not mix and form segregated superlattices, the the resulting pattern will simply be the sum of patterns for single-material superlattices. All patterns are plotted on the 2θ_Cu Kα_ scale and include instrumental intensity corrections (thin-film Lorentz polarization, instrumental broadening).^19^ See SI, section S4 for details.

In addition to our reported results, we present here simulated multilayer diffraction patterns for some stimulating case studies, comparing nanocrystals packed in different geometries, nanoplatelets of different thicknesses, and discussing possible results of coassembling two different materials (Figure 4; simulation conditions are discussed in the SI, section S4). These simulations aim to capture the general distribution and relative intensities of fringes, but their exact position is dictated by the superlattice periodicity Λ, which could only be estimated. As such, the fringe positions shall not be used to identify the superlattice, in contrast to the common practice of recognizing materials from their peak positions in a powder XRD pattern.

Figure 4a compares patterns for spherical Cs_4_PbBr_6_ nanocrystals packed in different geometries (simple cubic, BCC, FCC, and HCP). The nanocrystal size, orientation, and interparticle distance are kept constant for comparison. Each packing geometry results in a different Λ, which is reflected in the fringe periodicity *Δq*. Note that for the FCC, BCC, and HCP packings, the measured Λ is half of the superlattice unit cell because each includes two nanocrystal layers. Figure 4b shows instead patterns calculated for CdSe nanoplatelets of different thicknesses, increasing from top to bottom and separated by a constant interparticle distance. As the thickness increases, some of the diffracted intensity progressively localizes around 29° 2θ_Cu Kα_, where the (200) Bragg peak of sphalerite-CdSe would eventually form for thick nanocrystals.

Figure 4c addresses the coassembly of PbS and CsPbBr_3_ nanocubes into superlattices where the two materials are randomly mixed or alternate in layers. These two cases would be very challenging to distinguish by GISAXS, as the overall superlattice periodicity and geometry are the same. However, multilayer diffraction can tell them apart by the periodicity of fringes at the first perovskite peak (Figure 4c, insets). Indeed, alternating the two materials effectively doubles the distance between perovskite nanocrystals, resulting in fringes twice as close to each other. A similar effect is seen in Figure 4d, where nanoplatelets of CsPbBr_3_ and PbS are coassembled. Again, the doubled superlattice periodicity results in the appearance of extra fringes in the 0–23° 2θ range (red arrows), which, however, disappear in the second part of the pattern. This is because CsPbBr_3_ and PbS have a similar diffraction profile in the 23–37° 2θ range, as seen by the two single-material patterns. Therefore, in that area of the pattern they behave as if they were the same material, thus virtually halving the superlattice periodicity.

## Beyond Lead Halide Perovskites

**Figure 5:**
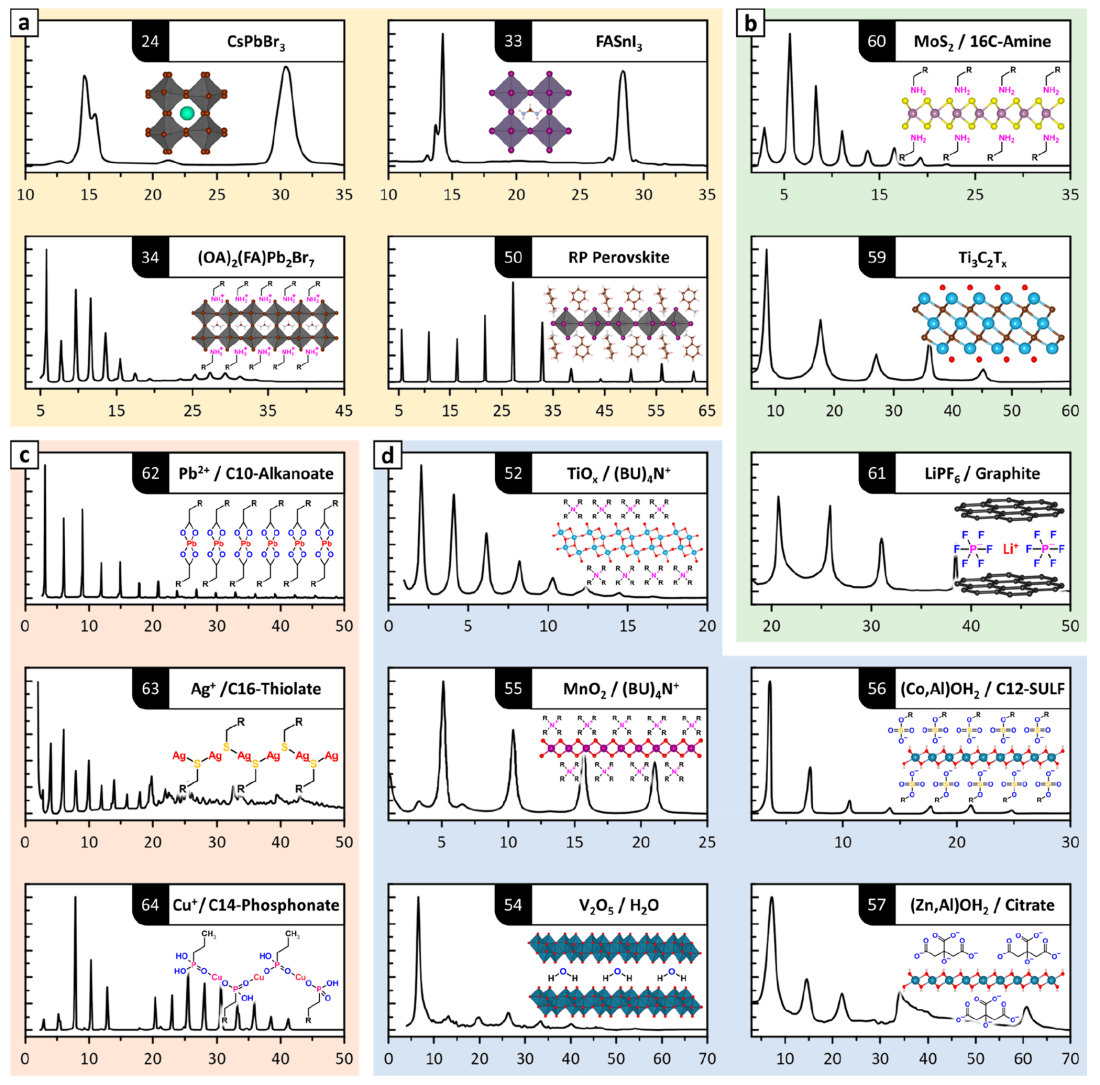
Literature patterns showing multilayer diffraction. Colors identify classes of materials. (a) Yellow: metal-halide perovskites. (b) Green: synthetic 2D materials. (c) Red: metal–organic precursors for colloidal syntheses. (d) Blue: metal oxides and hydroxides. Numbers within black labels indicate the corresponding reference. All patterns were digitized with WebPlotDigitizer^65^ and are plotted on the 2θ_Cu Kα_ scale. Abbreviations: OA = oleylamine; RP = Ruddlesden–Popper; *T* = (F, OH, O); BU = butyl-; SULF = sulfate. Data are taken from refs (24), (33), (34), (50), (52), (54−57), and (59−64).

Following the simulations we just discussed, we encourage the reader to think beyond lead halide perovskites, because multilayer diffraction is by no means a phenomenon exclusive to them. Indeed, a literature search reveals multilayer diffraction effects in a variety of materials (Figure 5): apart from halide perovskites,^24,33,34,50^ interference fringes are seen for metal oxides and hydroxides,^51−57^ for synthetic two-dimensional materials such as MXenes, metal dichalcogenides, and intercalated graphite,^58−61^ and also for metal–organic salts often used as precursors in the synthesis of nanomaterials.^62−64^ All of these systems, and in general any material that is prone to be self-assembled, exfoliated, or stacked, are suitable building blocks for constructing highly ordered structures. It is in the hands and minds of researchers to recognize multilayer diffraction and use it to empower their research.

We emphasize that, before being a characterization tool, multilayer diffraction is first and foremost an intrinsic behavior of the sample. Therefore, it can be exploited only when naturally present, just like photoluminescence spectroscopies are useful only on intrinsically luminescent samples. When applicable, multilayer diffraction analysis is highly complementary to established superlattice characterization techniques. For example, multilayer diffraction excels in quantifying the positional disorder of nanocrystals, while GIWAXS is highly sensitive to nanocrystal tilting.

Compared to GISAXS, multilayer diffraction enables a more accurate quantification of Λ, σ_*L*_, and small variations thereof because information is extracted at higher angles and from multiple fringes. Moreover, multilayer diffraction is sensitive to the crystal structure of particles, and because it is based on the interference between neighboring nanocrystals, it conveys information about their surroundings. Hence, multilayer diffraction is a valid tool for systems composed of two or more mixed nanomaterials, where their relative positioning at the local level would be far from obvious from small-angle diffraction experiments. Binary and ternary superlattices based on perovskite nanocrystals could be suitable samples for testing these predictions.^66,67^ On the other hand, multilayer diffraction lacks the capability, typical of GISAXS, to unambiguously identify the packing geometry in 3D superlattices and 2D monolayer nanocrystal assemblies, although in the first case this can be inferred indirectly from the superlattice periodicity as illustrated in Figure 4a.

To conclude, multilayer diffraction does not require specialized instrumentation, as it can be measured on any θ:2θ laboratory-grade diffractometer. Moreover, its high information density, stemming from combining atomic- and nanometric-scale information in a single experiment, makes it especially suitable for in situ and operando experiments, where speed and simplicity become crucial. Nevertheless, there is room for increasing the versatility of multilayer diffraction even further. One way would be observing interference patterns in other experimental geometries than θ:2θ scans performed on flat macroscopic samples. The first steps in this direction have been recently made by recognizing and modeling multilayer diffraction in liquid suspensions of CsPbBr_3_ assemblies investigated by a total scattering approach.^26^ Similarly, multilayer diffraction might be studied at the single-aggregate level by microdiffraction experiments, and might provide information on the horizontal structure of superlattices if collected in transmission geometry. Finally, it will be crucial to develop versatile and user-friendly multilayer diffraction software for the routine and high-throughput analysis of nanocrystal and nanoplatelet solids, which would help in establishing the method among the colloidal nanocrystal community.

## Supporting Information Available

Comparison of symmetric θ:2θ scan and grazing-incidence diffraction geometries; impact of nanocrystal morphology and superlattice thickness on multilayer diffraction; and details of simulations shown in Figure 4 (PDF)
