# Supplementary material for: Collective Diffraction Effects in Perovskite Nanocrystal Superlattices
Source: Acc Chem Res. 2022 Dec 19;56(1):66–76. doi: 10.1021/acs.accounts.2c00613 (PMC9813911; doi:10.1021/acs.accounts.2c00613)
Supplement: Supplementary file 1 — ar2c00613_si_001.pdf [file ar2c00613_si_001.pdf]

Supporting Information for

Collective Diffraction Effects in Perovskite Nanocrystal Superlattices

Stefano Toso,[a,b] Dmitry Baranov,[a] Umberto Filippi,[a,b] Cinzia Giannini,[c] Liberato Manna[a]*

a) Department of Nanochemistry, Istituto Italiano di Tecnologia, Via Morego 30, 16163 Genova, Italy

b) International Doctoral Program in Science, Università Cattolica del Sacro Cuore, 25121 Brescia, Italy

c) Istituto Di Cristallografia – Consiglio Nazionale delle Ricerche (IC–CNR), I-70126 Bari, Italy

S1. θ:2θ symmetric scan *vs* grazing incidence geometries

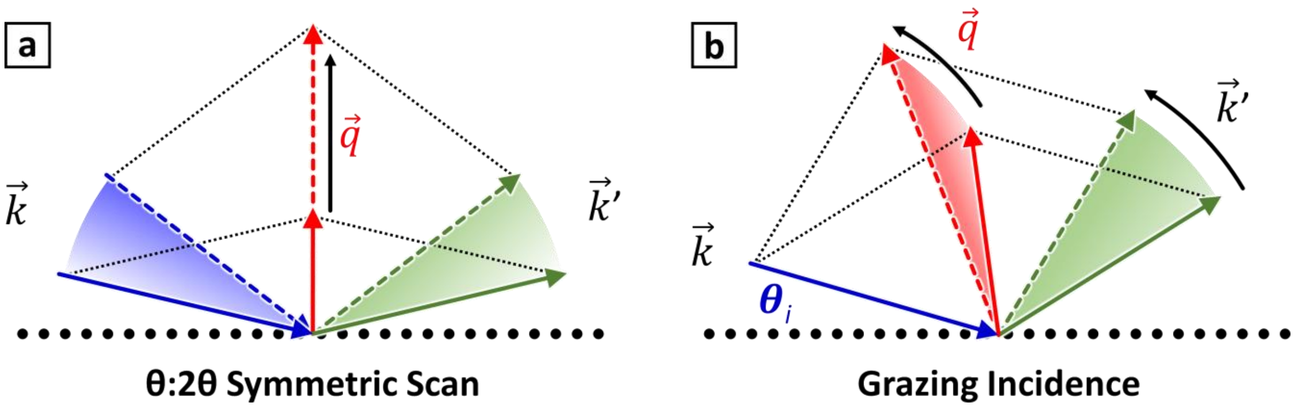

Figure S1. Scattering geometries compared. a) θ:2θ out-of-plane symmetric scan geometry adopted for Multilayer Diffraction experiments. b) Grazing incidence geometry typical of GISAXS and GIWAXS experiments. Here, $\vec{k}$ and $\vec{k}'$ are the wave vectors of the incident and scattered radiation, $\vec{q}$ is the scattering vector, and θ_i is the fixed angle of incidence adopted in grazing-incidence geometries (here depicted wider than in reality for illustration purposes).

Figure S1 compares the θ:2θ out-of-plane symmetric scan geometry adopted for Multilayer Diffraction experiments with the fixed-angle grazing incidence geometry adopted for GISAXS and GIWAXS experiments. As indicated by the variation in length of the red arrow as we move from a low-angle (solid lines) to a high-angle (dashed lines) data acquisition, both geometries can scan a range of $|\vec{q}|$ values. The main difference is that in the θ:2θ geometry the scattering vector $\vec{q}$ remains perpendicular to the substrate all throughout the experiment, while in the grazing incidence geometry this is never the case.

This is an important difference, because computing the diffracted intensity requires calculating the scalar product between the scattering vector $\vec{q}$ and the spatial position of scatterers (atoms or unit cells, see Equations 1-3 in the Main Text). Therefore, in a θ:2θ symmetric scan the horizontal coordinates of scatterers become irrelevant, and the sample can be described as a stack of planes with no specific in-plane structure. Conversely, in grazing-incidence experiments the horizontal coordinates of scatterers can never be neglected, making the quantitative prediction of diffracted intensity much more challenging.

S2. Impact of nanocrystal morphology on Multilayer Diffraction

As discussed in the Main Text, the morphology of nanocrystals mainly impacts their Multilayer Diffraction analysis by determining whether they are thick enough to be properly described by unit cells, in which case they are considered "*isotropic*" nanocrystals, or they are instead so thin to require an atomistic description of their structure, in which case they are considered nanoplatelets. The actual shape of nanocrystals (cubes, spheres, octahedra, etc.) has instead a rather small influence on their scattering factor, as F_{NC} is much more affected by the number of unit cells that compose the particle rather than by their spatial distribution, that is the particle shape.

Figure S2 illustrates this concept by comparing the diffraction profiles of individual $CsPbBr_3$ nanocubes and nanospheres (Figure S2a), and their simple-cubic superlattices (Figure S2b), simulated by keeping constant the volume of nanoparticles (7.5 nm edge cube and 9.3 nm diameter sphere, V ≈ 420 nm^3) and the superlattice structural parameters (nanocrystal orientation, Λ, and σ_L). The nanocrystal morphology is described by the factor $T_{(hkl,n)}$, which is the number of unit cells per lattice plane. This factor can be calculated for any given shape (Figure S2c). Simulations show that the main difference between cubic and spherical nanocrystals is the presence, or absence, of low-intensity ripples at the sides of the main Bragg peaks. These ripples are related to the steepness of the electron density gradient between the center and the upper/lower limits of the nanocrystal. However, such ripples would be difficult to observe experimentally due to their low intensity and due to the broadening induced by a non-zero nanocrystal size dispersion and instrumental contributions. Yet, the nanocrystals morphology might have an indirect impact on the Multilayer Diffraction profile of their superlattices by deciding the packing geometry (such as simple cubic, BCC, FCC, HCP, etc.), as illustrated in Figure 4 of the Main Text.

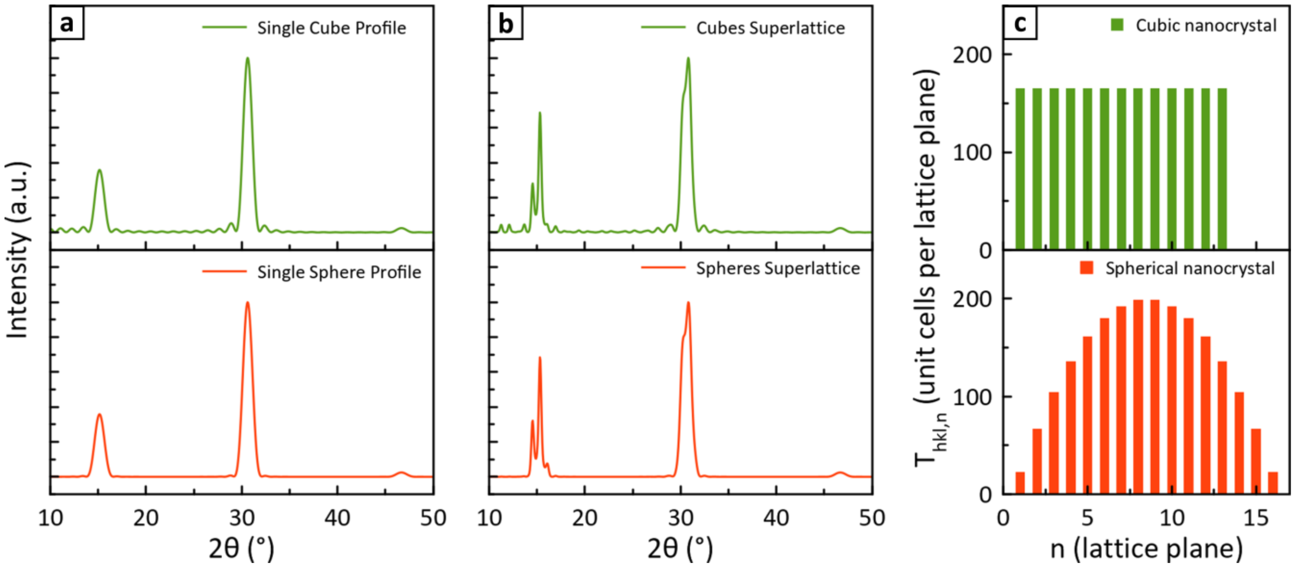

Figure S2. Impact of nanocrystal morphology on Multilayer Diffraction. a) Simulated diffraction patterns of a single $CsPbBr_3$ nanocube (green) and a single $CsPbBr_3$ nanosphere (red) having approximately the same volume. b) Simulated Multilayer Diffraction patterns of two simple cubic superlattices composed of the same $CsPbBr_3$ nanocubes and nanospheres simulated in panel (a). c) $T_{hkl,n}$ shape factors for the cubic and spherical $CsPbBr_3$ nanocrystals simulated in panels (a) and (b). The $T_{hkl,n}$ factor expresses the shape of a nanocrystal by cutting its volume into n slices, one per each plane belonging to the (hkl) family, and converting the volume of each slice into the corresponding number of unit cells.

S3. Influence of superlattice thickness on Multilayer Diffraction

In principle, thick superlattices are not required to observe Multilayer Diffraction. Figure S3a demonstrates that superlattices as thin as 2 nanocrystals can produce interference fringes, and the thickness of the self-assembled domains ceases to have a detectable effect on the diffraction profile above ~10 nanocrystals.

What is crucial instead is that the overall superlattice periodicity stays consistent within each self-assembled domain (which means that σ_L is locally low), but also between all domains within the same sample (which can be visualized as a globally low σ_L, although only as a semiquantitative analogy). Indeed, if that were not the case, all domains would produce interference fringes in slightly different positions. Then, measuring a vast area of the sample would result in these differences smoothing out the diffraction profile, thus yielding a pattern identical to that of individual and non-interfering nanocrystals.

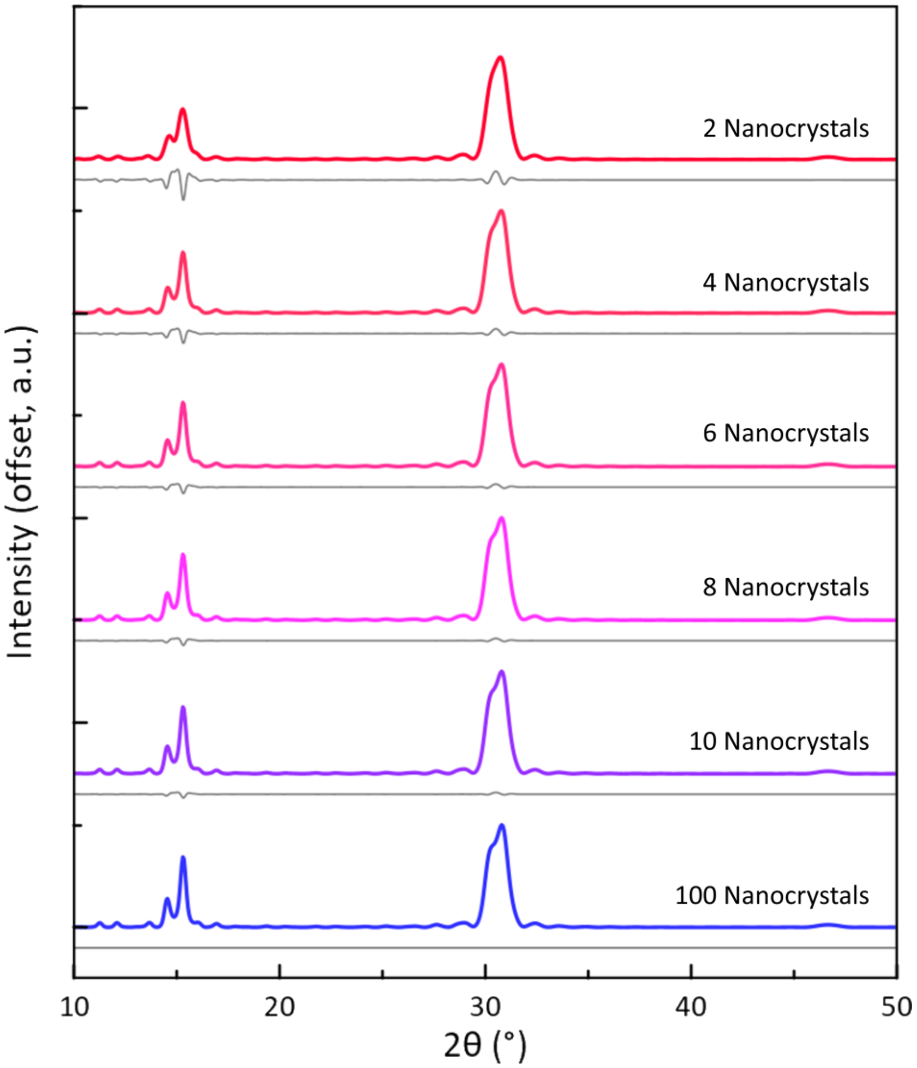

Figure S3. Impact of the superlattice thickness on Multilayer Diffraction. Simulated diffraction profiles of $CsPbBr_3$ nanocubes superlattices of different thicknesses, highlighting how superlattices as thin as 2 nanocrystals can already display interference fringes, and the diffraction profile becomes virtually insensitive to the superlattice thickness above ~ 10 Nanocrystals. Grey lines indicate the difference between each profile and the reference 100 nanocrystals-thick superlattice profile.

S4. Simulation conditions for Figure 4

The superlattice periodicity values (*Λ*) of Cs_4PbBr_6 nanocrystals superlattices were estimated by assuming a hard packing of perfect spheres with total diameter (*D*+*L*), where *D* is the diameter of the nanocrystal and *L* is the surface-to-surface distance between two neighboring nanocrystals. The following geometrical relations were used for each of the packing geometries:

$$\Lambda_{S.Cubic} = D + L$$

$$\Lambda_{BCC} = \frac{2}{\sqrt{3}} \cdot (D + L)$$

$$\Lambda_{FCC} = \frac{2}{\sqrt{2}} \cdot (D + L)$$

$$\Lambda_{HCP} = \sqrt{\frac{8}{3}} \cdot (D + L)$$

Table S1. Simulation parameters for Figure 4. "Orientation" indicates the planes parallel to the substrate. Surface terminations are specified only for nanoplatelets, as the unit-cell based description of nanocrystals makes such level of atomistic detail inaccessible. When *Λ* values are indicated as 2×number, it means that the actual superlattice unit cell parameter is double the layer center-to-center distance, which is the actual value provided to the simulation algorithm.

Plot	Material	Orientation	Shape	Thickness [nm]	Termination	L [Å]	σ_L [Å]	Packing Geometry	Λ [Å]
a1	Cs_4PbBr_6	(012)	Sphere	9.09	---	34	1.0	S. Cubic	124.9
a2	Cs_4PbBr_6	(012)	Sphere	9.09	---	34	1.0	BCC	2×72.1
a3	Cs_4PbBr_6	(012)	Sphere	9.09	---	34	1.0	FCC	2×88.3
a4	Cs_4PbBr_6	(012)	Sphere	9.09	---	34	1.0	HCP	2×102.0
b1	CdSe	(100)	Platelet	0.43	Cd	40	0.5	Stack	44.3
b2	CdSe	(100)	Platelet	1.29	Cd	40	0.5	Stack	52.9
b3	CdSe	(100)	Platelet	2.15	Cd	40	0.5	Stack	61.5
b4	CdSe	(100)	Platelet	3.01	Cd	40	0.5	Stack	70.1
c1	$CsPbBr_3$	(100)	Cubes	7.59	---	34	1.0	S. Cubic	109.9
c2	$CsPbBr_3$	(100)	Cubes	7.59	---	34	1.0	S. Cubic, alternated	110.5
	PbS	(100)	Cubes	7.71	---				
c3	$CsPbBr_3$	(100)	Cubes	7.59	---	34	1.0	S. Cubic, mixed	2×110.5
	PbS	(100)	Cubes	7.71	---				
c4	PbS	(100)	Cubes	7.71	---	34	1.0	S. Cubic	111.1
d1	$CsPbBr_3$	(100)	Platelet	1.18	$R\text{-}NH_3Br$	37	0.5	Stack	48.8
d2	$CsPbBr_3$	(100)	Platelets	1.18	$R\text{-}NH_3Br$	36.9	0.5	Stack, alternated	2×48.8
	PbS	(100)	Platelets	1.21	PbS				
d3	$CsPbBr_3$	(100)	Platelets	1.18	$R\text{-}NH_3Br$	36.9	0.5	Stack, mixed	48.8
	PbS	(100)	Platelets	1.21	PbS				
d4	PbS	(100)	Platelets	1.21	$R\text{-}NH_3Br$	36.8	0.5	Stack	48.8
